# Diagnostic accuracy of iodine quantification and material density imaging with rapid Kilovoltage-switching DECT for small hyperattenuating renal lesions

**DOI:** 10.1007/s00261-025-04964-2

**Published:** 2025-05-02

**Authors:** Shanigarn Thiravit, Adisa Moleesaide, Rathachai Kaewlai, Chayanit Limsakol, Arjin Maneegarn, Arissa Phothisirisakulwong, Phakphoom Thiravit

**Affiliations:** Division of Diagnostic Radiology, Department of Radiology, Faculty of Medicine Siriraj Hospital, Mahidol University, Bangkok, Thailand

**Keywords:** DECT, spectral CT, Renal mass, Renal cyst, Iodine, Rapid-kilovoltage switching

## Abstract

**Objectives:**

To assess accuracy of MDI and iodine quantification in distinguishing enhancing renal masses from hyperattenuating cysts, compared with conventional attenuation measurements, given that differentiation between these entities can influence follow-up imaging strategies and surgical decision-making, and to investigate the optimal threshold of iodine concentration using rapid kilovoltage-switching DECT (rsDECT).

**Materials and methods:**

Retrospective study enrolled 126 renal lesions 1–4 cm in size with 10–70 attenuation on pre-contrast CT in patients who underwent rsDECT during the portovenous phase. Two reading sessions (true unenhanced (TUE) + post-contrast (PC) + MDI images versus MDI only images) for the visual assessment of renal mass enhancement were done (with at least 1-month time gap). Measurement of attenuation and iodine concentration within each renal lesion was recorded. Diagnostic accuracies and a threshold of each quantitative parameters were evaluated. Final diagnosis of renal lesions was based on pathological or imaging criteria.

**Results:**

Accuracy of MDI images were 90.5% with TUE + PC + MDI and 88.9% with MDI only. AUC of VUE HU, TUE HU, PC HU, PC VUE HU, PC-TUE HU, absolute and normalized iodine concentration were 0.87, 0.82, 0.96, 0.95, 0.96, 0.97 and 0.95 (all *p* < 0.001). The optimal absolute iodine concentration threshold was 1.6 mg I/mL, with 91% sensitivity and 92% specificity. This threshold outperformed 0.5 mg I/mL showing 100% sensitivity, 29% specificity) and 2.0 mg I/mL showing 71% sensitivity, 97% specificity.

**Conclusion:**

In characterization of a small (< 4 cm) hyperattenuating renal lesion identified on abdominal CT, post processing MDI with iodine quantification has better or comparable accuracy to attenuation measurement and the specificity of iodine concentration using rsDECT improves with a threshold higher than 0.5 mg I/mL. This could enhance diagnostic workflows for renal lesion assessment using MDI and offer the potential to omit TUE scanning, thereby reducing patient radiation exposure.

**Graphical abstract:**

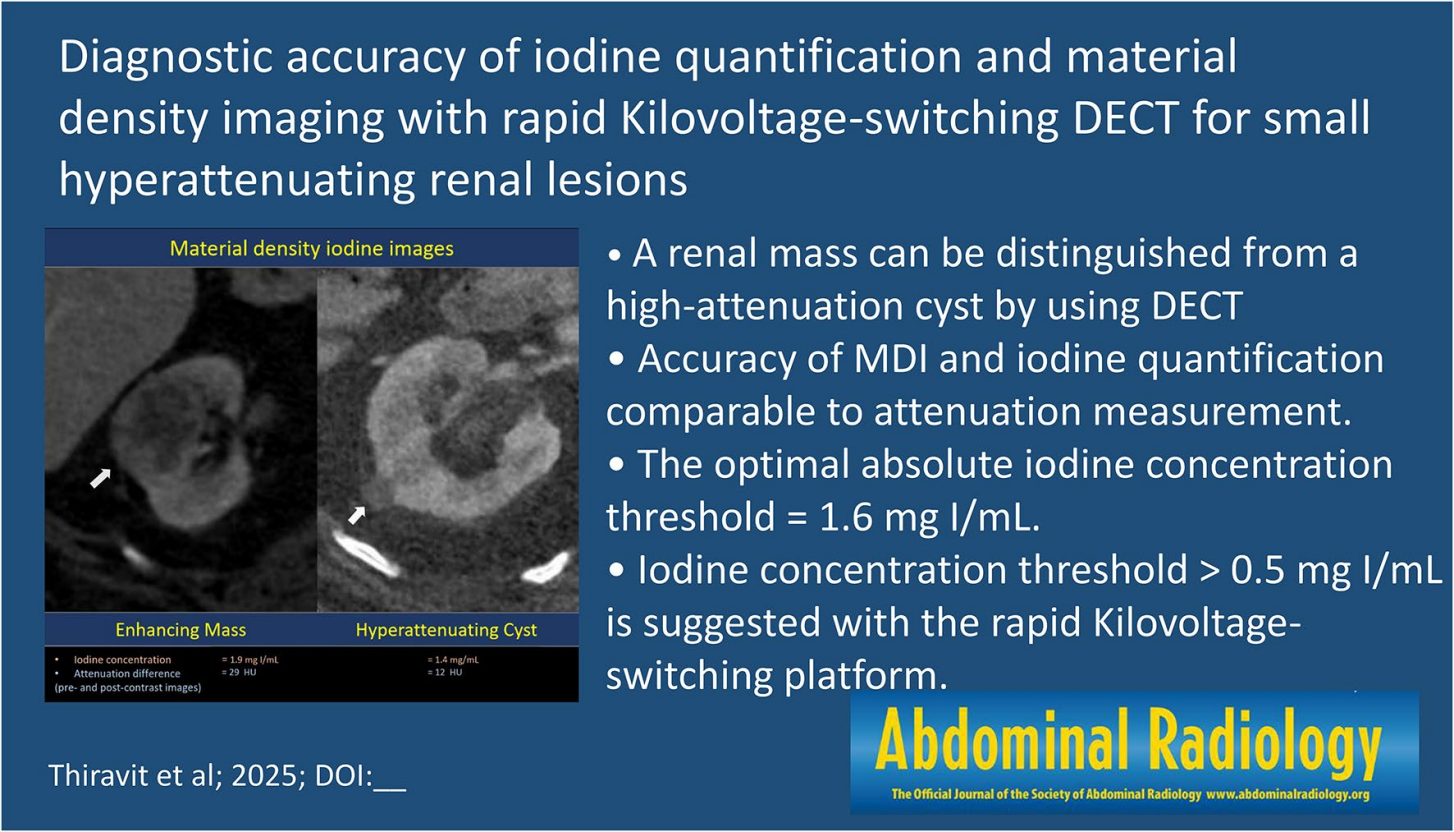

## Introduction

Renal cysts are typically identified as uniformly low-density lesions within the renal tissue, with attenuation values ranging between − 10 and 20 Hounsfield units (HU) on a non-contrast CT scan [1, 2]. Any lesion that exhibits an attenuation value higher than this range is considered hyperattenuating and can be indeterminate. Although many of these lesions are benign—such as hemorrhagic or proteinaceous cysts—they can also represent solid renal masses [1]. According to the previous report, indeterminate renal lesions are identified in up to 21% of initial imaging studies [3]. Of these cases, over one-quarter of patients underwent immediate treatment without additional imaging or renal mass biopsy, with benign pathology identified in approximately 10% [1, 3]. These findings highlight the importance of accurately assessing whether a hyperattenuating lesion demonstrates internal enhancement, which may indicate a solid component. When no enhancement is present, further imaging or unnecessary surgical intervention can potentially be avoided [1].

The Bosniak classification, version 2019, defines enhancement as present when there is an attenuation difference of 20 HU or greater before and after intravenous contrast administration within the mass [2]. In alignment with the ACR white paper, additional imaging for further characterization is recommended for any incidental homogeneous renal lesions with attenuation greater than 20 HU on contrast-enhanced CT [4]. However, some renal cell carcinomas (RCCs), such as the papillary subtype, may show indeterminate range enhancement (10–20 HU) or no enhancement (< 10 HU) [2]. These certain lesions may be missed by the conventional measurement method.

Dual-energy CT (DECT) is a promising modality that provides advanced tissue characterization by utilizing two distinct energy levels—high energy (140–150 keV) and low energy (50–80 keV). This technique allows for the acquisition of data at different energy levels, which can be post-processed to isolate specific materials within the body. For example, iodine can be visualized through material density iodine (MDI) images or iodine mappings. These MDI images can help differentiate a renal mass from a cyst by identifying iodine-containing voxels, which indicate enhancement, and by measuring the internal iodine levels [5, 6]. The ability to quantify iodine within a lesion using DECT offers a more advanced approach than observing attenuation differences between the pre- and post-contrast phases for renal mass assessment. MDI assessment for renal mass evaluation appears feasible for general radiologists and does not necessarily require additional expertise [5]. Previous studies have shown that iodine quantification can effectively distinguish between enhancing and non-enhancing renal lesions with high sensitivity and specificity [7–10]. However, optimal iodine density thresholds can vary depending on the DECT platform used (e.g., dual-source, rapid kilovoltage-switching (rsDECT), or dual-layer) [11]. This variability can be attributed in part to differences in algorithms used for material decomposition and iodine quantification across platforms [11, 12]. In addition to scanner-dependent factors, variability may also result from intra-patient differences, such as variations in cardiac output and physiological iodine uptake, as well as study protocol differences, including contrast injection protocols and the timing of image acquisition [11, 12].

In this study, we retrospectively analyzed small hyperattenuating renal lesions in a patient who underwent rsDECT imaging. This study aimed to assess the diagnostic performance of MDI images and iodine quantification derived from DECT in distinguishing enhancing renal lesions from hyperattenuating renal cysts compared with attenuation measurements and to find the optimal iodine threshold specific to the rsDECT platform.

## Materials and methods

### Study population

**Fig. 1:**
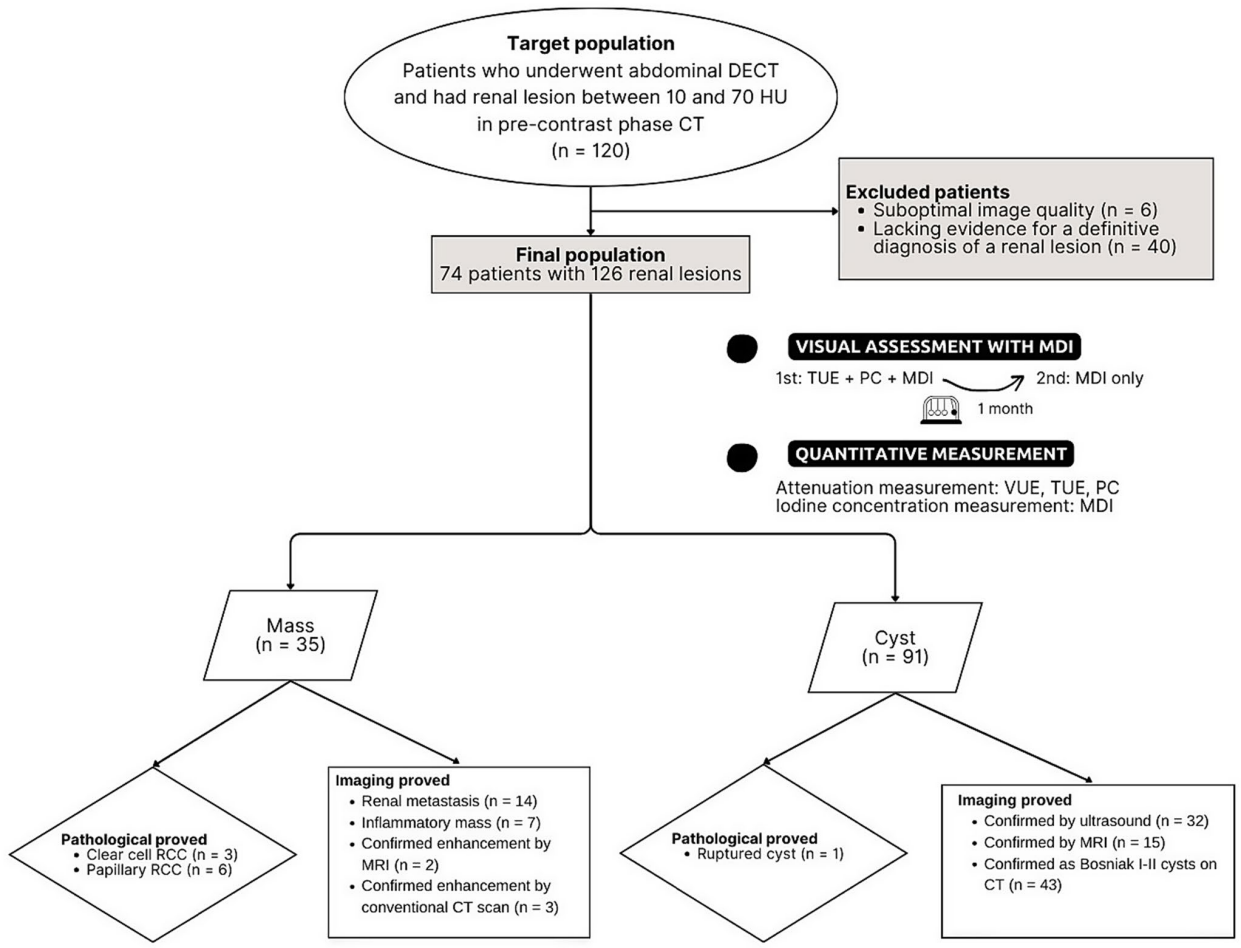
Flowchart of study population and image interpretation. DECT = dual-energy CT, Hounsfield units = HU, TUE = True unenhanced image, PC = Post contrast images, MDI = Material density iodine images, VUE = Virtual unenhanced image, RCC = Renal cell carcinoma

This retrospective study was conducted at a single tertiary center with approval from our Institutional Review Board (COA no. Si 205/2023). Population was included a list of patients who found to have a renal lesion between 10 and 70 HU in pre-contrast phase CT with a size of 1–4 cm on routine abdominal DECT exams performed during the portovenous phase at our hospital between May 2019 and August 2022. In our study, we defined a high-attenuating lesion as any lesion with attenuation greater than 10 HU on a non-contrast image, as this may indicate the presence of materials such as hemorrhage or high-protein content, rather than simple fluid inside the lesion. We did not include lesions with attenuation greater than 70 HU, as these are typically considered hemorrhagic cysts and classified as Bosniak Classification, version 2019, class II [2]. If there were more than 1 lesion per kidney, the largest two lesions per kidneys were selected to include in our study. The following exclusion criteria were if there was suboptimal image quality or insufficient confirmatory evidence for a definitive diagnosis of a renal lesion or underlying autosomal dominant polycystic kidney disease. A total of 74 patients with 126 renal lesions was enrolled in this study (Fig. 1).

### Imaging protocol

All the CT examinations were performed using a rsDECT scan (CT Revolution apexTM, GE healthcare) which switches between 80 kVp and 140 kVp in 0.5 msec. CT acquisition parameters as follow; tube current, 275 mAs; Pitch, 0.992; rotation time, 0.5 s; collimation, 80 × 1.5 mm; ATCM, off; iterative reconstruction, 40% ASIR-V. Reconstruction slice thickness and interval were 1.25 mm for all devices. Calibration for attenuation values was routinely performed on every morning of weekdays. The abdominal CT protocol included true unenhanced (TUE) images and DECT images performed during the portovenous phase at 90 s after start of contrast media injection. Subsequently, the contrast-enhanced DE datasets were transferred to workstation equipped with Gemstone spectral imaging viewer software (AW server2, release 5.5, GE healthcare). Reconstructed virtual unenhanced images (VUE), MDI images displaying as the axial grayscale image (as pixels of increased iodine signals appearing brighter in gray scale) of 1.25 mm slice thicknesses were sent to PACS system.

### Image interpretation and reading sessions

Third year radiology residents who did not perform the image analysis reviewed images and bookmarked a renal lesion on post contrast images in PACS system. Two board-certified body imaging radiologists (with 12 years and 5 years of experiences) blinded to the final diagnosis of a renal lesion independently determined enhancement within a renal lesion on the post-processing images by visual assessment. Two image reading sessions were done with at least 1-month time gap. On the first reading session, a combination of conventional TUE + post-contrast (PC) + MDI images was used to review which this session represented a routine use of MDI images as an adjunct to conventional images on image interpretation. For the second session, we examined a renal mass by using only MDI images. This represented a use of MDI images as a standalone sequence. For both sessions, each renal lesion was evaluated by visual assessment for iodine presence or absence and readers also had to score a confident level which was classified into 3 categories: (1) very confident, (2) somewhat confident and (3) not confident.

### Region of interest (ROI) measurement of renal mass enhancement

Attenuation values in HU of each renal lesion on TUE, VUE, and PC images was measured by drawing a ROI encompassed as much of the lesion’s surface as possible. Values were used to calculate the attenuation difference between PC and TUE images (PC-TUE), as well as between PC and VUE images (PC-VUE).

Iodine concentration value of each renal lesion was also measured by drawing a ROI on MDI images then recorded in mg I/mL. The iodine concentration value of the aorta at the level of the renal artery was also recorded and used to calculate the normalized iodine concentration (the absolute iodine concentration value of a renal lesion divided by the iodine concentration value of the aorta). The normalized iodine concentration value may provide a solution for cases with improper post-contrast phases and reduce intermanufacturer variability.

### Final diagnosis

Clinical reference standard was evaluated by a third-year radiology resident who had an access to patient’s medical and imaging records. A renal lesion was classified as an enhancing renal mass if it met a pathological criteria obtained from surgery or tissue biopsy, or an imaging criteria as follow; (1) size changes of the renal lesion on follow-up CT scans alongside with the history of non-renal cancer and patterns indicative of metastasis, pointed to a potential renal metastasis, (2) size changes of the renal lesion on follow-up CT scans alongside with the clinical and treatment history of inflammatory noncancerous condition, indicative of an inflammatory mass, (3) MRI confirming enhancement characteristics included any solid enhancing lesions or thick perceptible enhancing septa or wall, or solid enhancing components (Bosniak classification 2019 category III-IV), (4) unequivocal sign of enhancement on conventional CT scan defined as attenuation value difference between TUE and PC images greater than 20 HU in the lesion failed to fit the above items 1–3 for malignant.

A renal lesion was classified as a cyst if it met a pathological criterion obtained from surgery or tissue biopsy, or an imaging criterion that confirmed no enhancement as follow; (1) ultrasound confirming a simple cyst, (2) MRI confirming no enhancement in subtraction images with typical MRI signals for a simple cyst (hypointense on T1 and hyperintense on T2) or a proteinaceous/ hemorrhagic cyst (hyperintense on T1 and hypo/iso on T2), (3) CT confirming no enhancement defined as attenuation value difference between TUE and PC images less than 20 HU either with or without few thin septa (Bosniak classification 2019 category II) in the lesion failed to fit the above items 1–2 for benign.

### Statistical analysis

The categorical baseline characteristics as gender was presented as number or percentage and normal continuous baseline characteristics as age, tumor size, and radiation dose were carried out by mean or median with standard deviation by cross tab analysis.

Diagnostic performance of each visual assessment method was calculated with cross tabulation with chi-square analysis and eventually compared. Confident scores of readers on image interpretation at each method were also reported as mean confident score which we grade confident scoring as follows: not confident = 1 point; somewhat confident = 2 point; very confident = 3 point. The reader agreement on interpretation of renal mass enhancement was quantified with Kappa coefficient as follows: poor agreement, 0.0-0.2; fair agreement, 0.21–0.40; moderate agreement, 0.41–0.60; strong agreement, 0.61–0.80; near complete agreement, 0.81-1.00.

For quantitative data analysis, the Mann-Whitney U test was used to compare attenuation values on VUE, TUE, PC, PC-TUE and PC-VUE images, iodine concentration values of enhancing lesions and hyperattenuating cysts, calculating the mean, median and standard deviation. Area under the curve (AUC) of each quantitative parameters was calculated by a receiver operating characteristic (ROC) analysis. The sensitivity and specificity of the pre-defined attenuation threshold on VUE and TUE HU at 20, PC HU at 30, PC-VUE HU and PC–TUE HU at 20, the fixed iodine concentration at 0.5 mg I/mL and 2 mg I/mL were computed. The optimal thresholds on VUE, TUE, PC, PC–VUE, PC–TUE, absolute and normalized iodine concentration were also determined by ROC curve analysis and selecting optimal sensitivity and specificity values by maximizing Youden J statistics. A p-value less than 0.05 was statistically significant. All statistical analyses were performed using SPSS software (version 28.0). Statistical significance was defined as a probability value of less than 0.05.

## Results

### Sample characteristics

There was a total of 74 patients with 126 renal lesions included in this study. The included patients consist of 36 females (48.6%) and 38 males (51.4%) with an average age of 67.6 ± 13.3 year. Final diagnosis was 35 enhancing masses and 91 hyperattenuating cysts.

Of 35 masses, 9 were established by pathological criteria (clear cell RCC; *n* = 3, papillary RCC; *n* = 6), 26 were established by imaging criteria (renal metastasis; *n* = 14, inflammatory mass; *n* = 7, confirmed enhancement by MRI; *n* = 2, and confirmed enhancement by conventional CT scan; *n* = 3). Of 91 hyperattenuating cysts, 1 was established by pathological proved as a ruptured cyst and the rest were established by imaging criteria (confirmed by ultrasound; *n* = 32, confirmed by MRI; *n* = 15, and confirmed as Bosniak I-II cysts on CT; *n* = 43) (Fig. 1).

The median of size of a renal mass (1.7 ± 0.80 cm; range, 1.0–3.3 cm) was comparable to a hyperattenuating cyst (1.6 ± 0.39 cm; range, 1.0–3.9 cm) (*p* = 0.814).

For a single energy unenhanced CT phase, the volume computed tomography dose index (CTDIvol) and the dose length product (DLP) were 8.79 ± 1.80 mGy and 431.88 ± 106.20 mGy-cm, respectively. A DE post contrast phase delivered a CTDIvol and DLP of 8.74 ± 2.28 mGy and 423.75 ± 125.71 mGy-cm, respectively.

### Visual assessment on MDI images between enhancing masses and hyperattenuating cysts

High diagnostic performance of image interpretation either using TUE + PC + MDI or MDI only to differentiate an enhancing mass from a hyperattenuating cyst was found for both readers. On the 1st reading session using TUE + PC + MDI, reader 1 and reader 2 yielded equal sensitivity of 94.3% but varying specificity of 89.0% and 94.5%, PPV of 76.7% and 86.8% and NPV 97.6% and 97.7%, respectively. On the 2nd reading session using only MDI images, the sensitivity of reader 1 was 94.3% similar to the sensitivity on the 1st reading session and for reader 2 was 97.1% being higher than the sensitivity on the 1st reading session. Specificity, PPV and NPV for reader 1 were 86.8%, 73.3% and 97.5% and for reader 2 were 82.4%, 68% and 98.7%, respectively (Table 1).

**Fig. 2:**
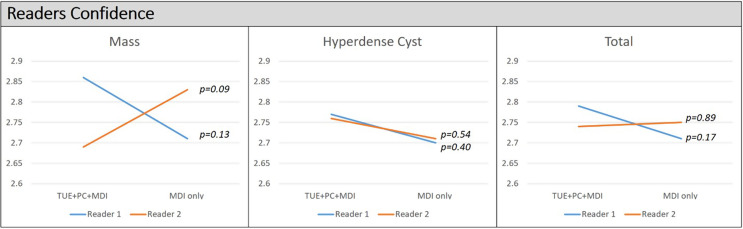
Confidence of readers for qualitative assessment using material density iodine images. TUE + PC + MDI = true unenhanced images + post-contrast images + material density iodine images. MDI only = material density iodine images only, P = p-value

There is no statistically significance difference of confidence score of each reader for image interpretation between two reading sessions (overall *p* > 0.05). However, the mean confidence score of reader 1 using TUE + PC + MDI was higher than the score using MDI only for interpretation of masses (2.86 ± 0.49 points versus 2.71 ± 0.57 points), hyperattenuating cysts (2.77 ± 0.45 points versus 2.70 ± 0.59 points) and total lesions (2.79 ± 0.46 points versus 2.71 ± 0.58 points). The mean confidence score of reader 2 using TUE + PC + MDI, compared with using MDI only for image interpretation revealed a lower score for masses (2.69 ± 0.47 points versus 2.83 ± 0.45 points), a slightly higher score for hyperattenuating cysts (2.76 ± 0.52 points versus 2.71 ± 0.52 points) and a slight lower score for total lesions (2.74 ± 0.51 points versus 2.75 ± 0.51 points) (Fig. 2).

The agreements between two readers for renal mass evaluation using TUE + PC + MDI and MDI only were near complete (k = 0.83) and strong (k = 0.78), respectively.

**Table 1:** Performance of qualitative assessment using material density iodine images on determination of enhancing renal masses

	Reader	Sensitivity	Specificity	PPV	NPV	Accuracy	*P*
TUE + PC + MDI	1	94.3 (80.8–99.3)	89.0 (80.7–94.6)	76.7 (64.6–85.6)	97.6 (91.3–99.4)	90.5 (83.9–95.0)	< 0.001
	2	94.3 (80.8–99.3)	94.5 (87.6–98.2)	86.8 (73.7–93.9)	97.7 (91.8–99.4)	94.4 (88.9–97.7)	< 0.001
MDI only	1	94.3 (80.8–99.3)	86.8 (78.1–93.0)	73.3 (61.7–82.4)	97.5 (91.1–99.3)	88.9 (82.0-93.8)	< 0.001
	2	97.1 (85.1–99.9)	82.4 (73.0-89.6)	68.0 (57.6–76.9)	98.7 (91.5–99.8)	86.5 (79.3–91.9)	< 0.001

Report in percentage with 95% CI in parenthesis

P-value are based on cross tabulation with chi square test analysis

TUE + PC + MDI = true unenhanced images + post-contrast images + material density iodine images

MDI only = material density iodine images only

### Attenuation and iodine concentration values between enhancing masses and hyperattenuating cysts

A statistically significant difference in median attenuation values (VUE, TUE, PC, PC-VUE and PC-TUE) and iodine concentration values (absolute and normalized) was found between masses and hyperattenuating cysts (all *p* < 0.001). The median attenuation value of masses was significantly higher than that of cysts on conventional measurements which was reported to be 35.8 HU versus 14.7 HU on VUE, 35.6 HU versus 19.3 HU on TUE, 80.9 HU versus 23.0 HU on PC, 44.6 HU versus 9.0 HU on PC-VNC and 50.1 HU versus 3.2 HU on PC-TNC, respectively. The median of absolute and normalized iodine concentration values of masses was higher than that of cysts which was 2.4 mg I/mL versus 0.7 mg I/mL for absolute concentration and 0.6 mg I/mL versus 0.1 mg I/mL for normalized concentration. Details of median, SD and range of attenuation and iodine concentration were reported in Table 2.

**Table 2:** Distribution and quantitative parameters of hyperattenuating renal lesions

		Lesion type						
		Mass (*n* = 35)			Hyperattenuating cyst (*n* = 91)			
		Median ± SD	Min	Max	Median ± SD	Min	Max	P
Attenuation value	VUE HU	35.8 ± 15.1	-4.7	77.8	14.7 ± 12.8	-4.3	56.6	< 0.001
	TUE HU	35.6 ± 9.3	13.0	53.0	19.3 ± 12.5	11.3	69.3	< 0.001
	PC HU	80.9 ± 27.2	33.3	142.0	23.0 ± 16.1	-5.7	84.4	< 0.001
	PC-VUE HU	44.6 ± 22.6	-	-	9.0 ± 9.9	-	-	< 0.001
	PC-TUE HU	50.1 ± 28.0	-	-	3.2 ± 8.0	-	-	< 0.001
Iodine concentration	Absolute (mg I/mL)	2.4 ± 1.1	1.0	5.1	0.7 ± 0.5	0.1	2.8	< 0.001
	Normalized	0.6 ± 0.2	0.0	0.9	0.1 ± 0.1	0.0	0.9	< 0.001

SD = Standard deviation

VUE = Virtual unenhanced image

TUE = True unenhanced image

PC = Post contrast images

The P value differences were calculated using the Mann–Whitney U test

### AUC and diagnostic thresholds of Attenuation and iodine concentration values

**Fig. 3:**
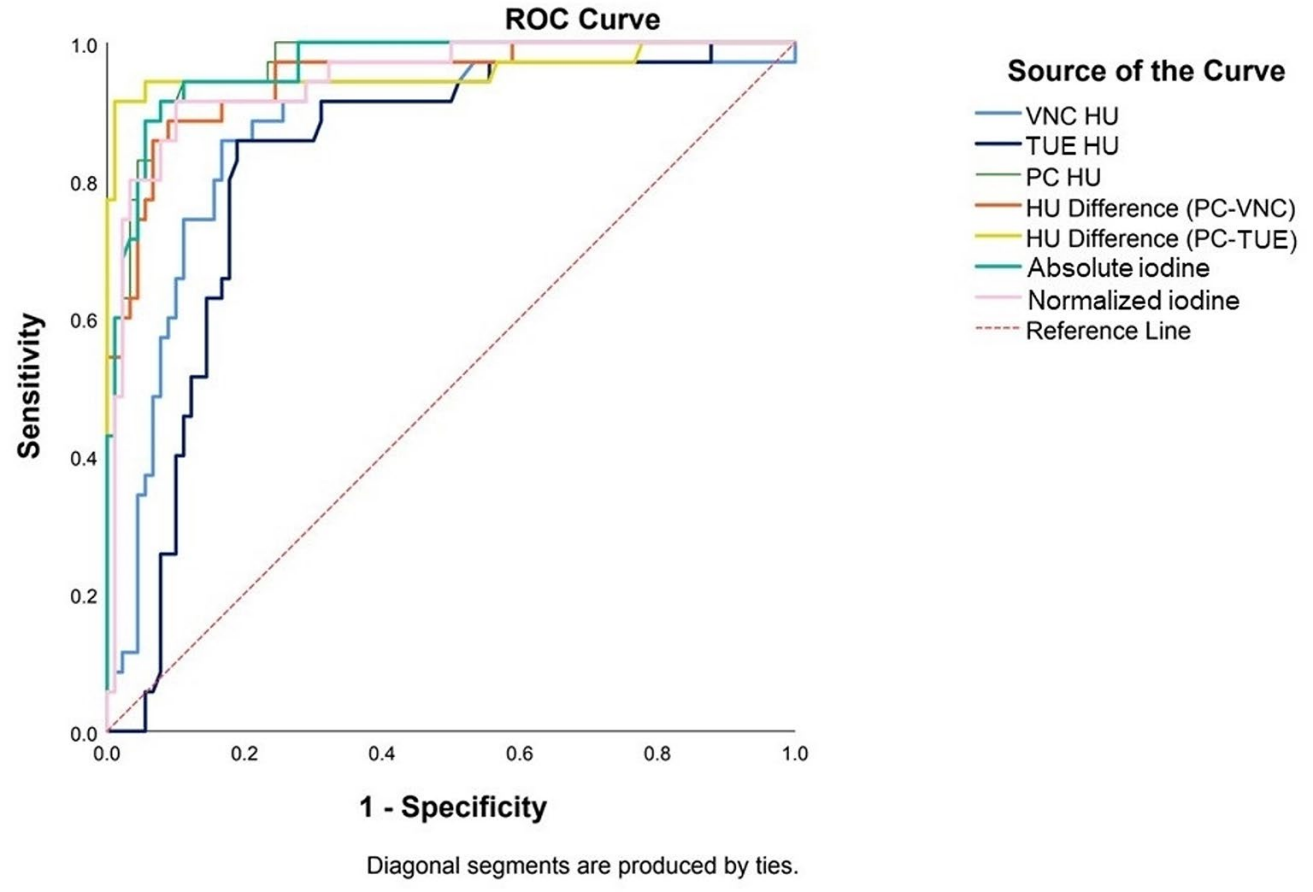
Area under the curve (AUC) values of thresholds of each parameter for distinguishing masses from hyperattenuating cysts by a receiver operating characteristic (ROC) analysis. AUCs of virtual unenhanced image (VUE HU) = 0.87, true unenhanced image (TUE HU) = 0.82, post contrast image (PC HU) = 0.96, attenuation difference threshold between PC and VUE images (PC-VUE HU) = 0.95 and attenuation difference threshold between PC and TUE images (PC- TUE HU) = 0.96, absolute iodine concentration = 0.97 and normalized iodine concentration = 0.95 (all *p* < 0.001)

The sensitivity and specificity with CIs and AUC with standard errors for the attenuation theshold of VUE HU, TUE HU, PC HU, PC-VUE HU, PC-TUE HU, absolute and normalized iodine concentration values for distinguishing enhancing masses from hyperattenuating cysts are summarized in Table 3; Fig. 3.

**Table 3:** Diagnostic performance of threshold of quantitative parameters to determine a solid renal lesion versus a hyperattenuating cyst

Parameter		Threshold	AUC	95% CI	Sensitivity	Specificity
Attenuation value	VUE HU	20	0.87	0.80, 0.95	0.91	0.70
	TUE HU	20	0.82	0.75, 0.90	0.91	0.55
	PC HU	30	0.96	0.94, 0.99	1.0	0.69
	PC-VUE HU	20	0.95	0.90, 0.99	0.89	0.84
	PC-TUE HU	20	0.96	0.91, 1.00	0.91	0.95
Iodine value	Absolute iodine (mg I/mL)	0.5	0.97	0.94, 0.99	1.0	0.29
		1.6	0.97	0.94, 0.99	0.91	0.92
		2.0	0.97	0.94, 0.99	0.71	0.97
	Normalized iodine	0.3	0.95	0.90, 0.99	0.91	0.90

VUE = Virtual unenhanced image

TUE = True unenhanced image

PC = Post contrast images

At pre-contrast images, AUCs of VUE HU and TUE HU were 0.87 (95% CI: 0.80, 0.95) and 0.82 (95% CI: 0.75, 0.90), respectively (*p* < 0.001). The pre-defined threshold of 20 HU on both VUE and TUE showed high sensitivity of 91% and 91% but low specificity of 70% and 55%. The optimal thresholds on both VUE and TUE were similarly reported to be at 22 HU with sensitivity and specificity of 91% and 75% on VUE and 89% and 69% on TUE.

At post contrast phase DECT, AUCs of PC HU was 0.96 (95% CI: 0.94, 0.99) (*p* < 0.001). The pre-defined threshold of 30 HU reported sensitivity of 100% but low specificity of 69% and the optimal threshold of 37 HU showed sensitivity and specificity of 94% and 84%.

AUC of PC-VUE HU and PC- TUE HU were 0.95 (95% CI: 0.90, 0.99) and 0.96 (95% CI: 0.91, 1.00), respectively (*p* < 0.001). The attenuation difference thesholds of 20 HU either on PC-VUE or PC-TUE were reported to be optimal and demonstrated better specificity with no decreased in sensitivity, compared with the threshold of VUE, TUE and PC attenuation. However, with the similar threshold, PC-TUE HU yielded higher sensitivity (91%) and specificity (95%), compared with PC-VUE HU (89 and 84%).

At MDI images, AUCs of absolute and normalized iodine concentration values were 0.97 (95% CI: 0.94, 0.99), and 0.95 (95% CI: 0.90, 0.99), respectively (*p* < 0.001). ROC analysis revealed the optimal thresholds to determine masses from hyperattenuating cysts to be of 1.6 mg I/mL for absolute iodine concentration with sensitivity and specificity of 91 and 92%, and of 0.3 mg I/mL for normalized iodine concentration with sensitivity and specificity of 91% and 90%. These showed similarly high diagnostic performances as the median attenuation difference on PC-TUE at 20 HU threshold. Prior studies reported the use of 0.5 mg I/mL and 2.0 mg I/mL as possible thresholds of absolute iodine concentration to determine enhancement in a renal lesion [9, 13, 14]. We found the threshold of 1.6 mg I/mL was more accurate than 0.5 mg I/mL and 2.0 mg I/mL and the associated sensitivities and specificities of those thresholds were reported in Table 3.

### Incorrectly classified renal lesions using the optimal thresholds of Attenuation difference and iodine concentration

**Fig. 4:**
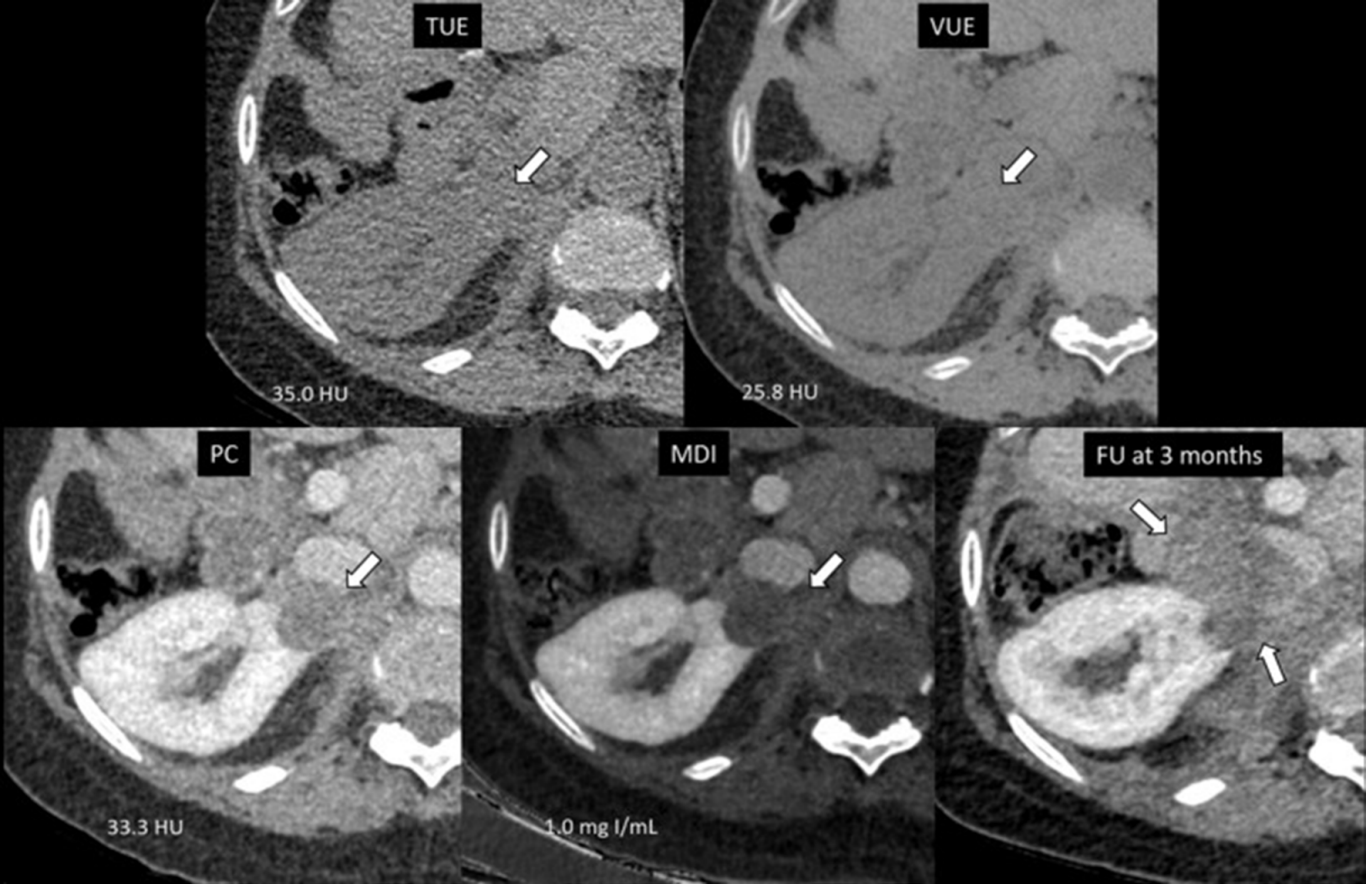
A 2.5 cm enhancing renal mass was incorrectly classified as a cyst due to attenuation differences of less than 20 HU between post-contrast and true unenhanced images, as well as an iodine concentration below 1.6 mg I/mL. This misclassification was likely due to internal necrosis within the mass. However, both readers were able to correctly identify it as a mass with high confidence upon visual assessment. The mass was pathologically confirmed as renal cell carcinoma and has shown size changes on follow-up CT scans. TUE = True unenhanced image, VUE = Virtual unenhanced image, PC = Post contrast images, MDI = Material density iodine images, FU = follow-up

**Fig. 5:**
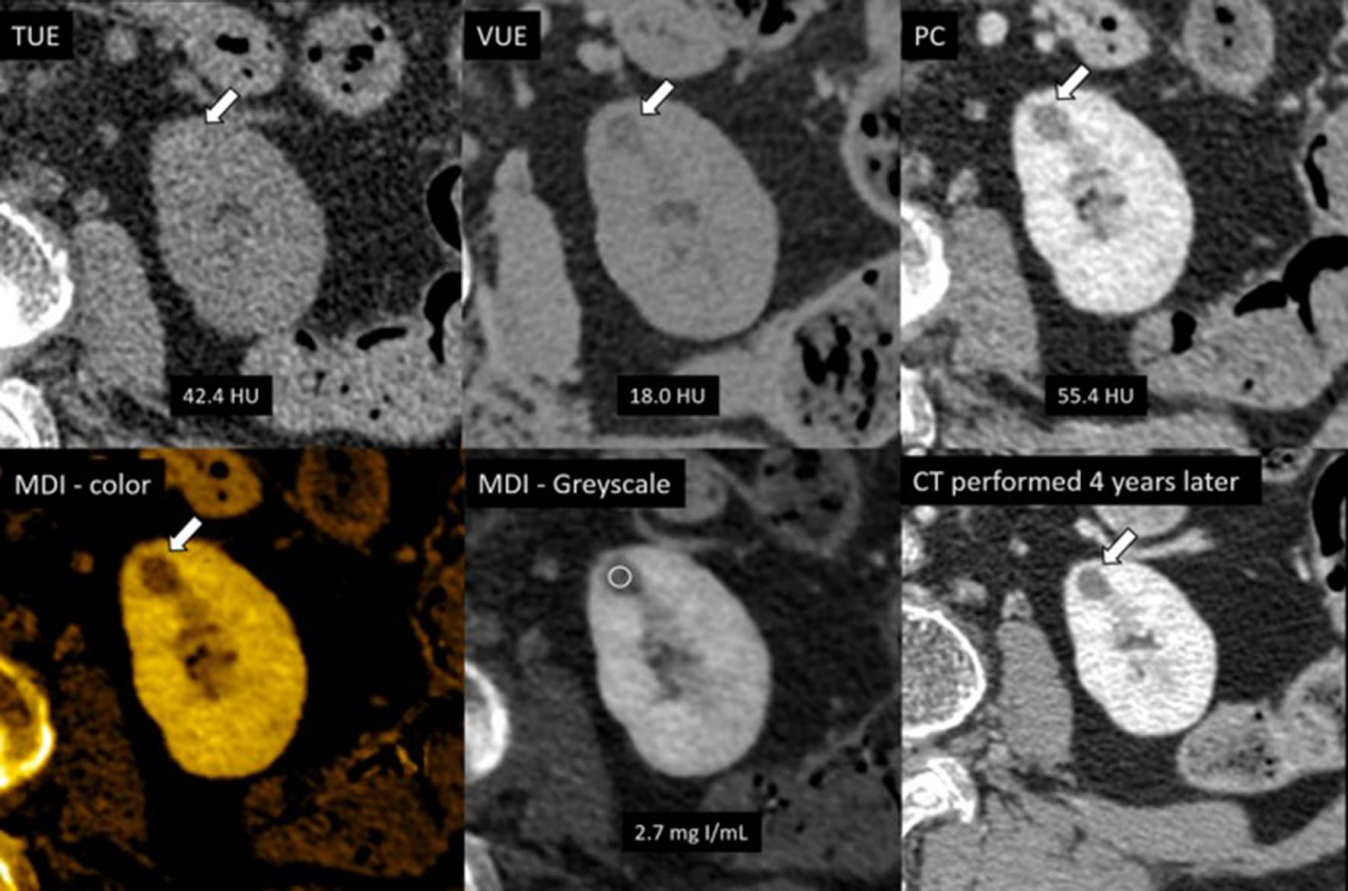
A 1.2 cm cyst with pseudoenhancement, misclassified as a mass based on iodine quantification (with an iodine concentration above the 1.6 mg I/mL cutoff). However, this lesion was correctly identified as a cyst on attenuation measurement and visual assessment of MDI images with high confident. This lesion was confirmed as a cyst by imaging criteria and has remained stable in size on CT imaging performed four years later. TUE = True unenhanced image, VUE = Virtual unenhanced image, PC = Post contrast images, MDI = Material density iodine images

Table 4 demonstrated number of renal lesions that were incorrectly classified when applying the optimal thresholds. In this study, 5/91 (5.5%) cysts were incorrectly classified as masses by demonstrating pseudoenhancement with an attenuation difference on PC-TUE more than 20 HU. Conversely, 3/35 (8.6%) masses were incorrectly classified as cysts as they had attenuation differences less than 20 HU (Fig. 4). When applying a cut-off of iodine concentration at 1.6 mg I/mL, we found that 6/91 (6.6%) cysts showed pseudoenhancement and were incorrectly classified as masses and 3/35 (8.6%) masses were incorrectly classified as cysts accordingly (Fig. 5). Comparing these results, both methods had a similar rate of false negatives but PC-TUE attenuation difference resulted in a slightly lower percentage of false positives for enhancing masses.

**Table 4:** Incorrectly classified renal lesions using optimal thresholds of Attenuation difference and iodine concentration

Parameters	Cysts with pseudoenhancement			Low-level enhancing masses		
	Number	Mean	Range	Number	Mean	Range
PC-TUE attenuation difference at 20 HU	5	22.98	21, 28.3	3	6.3	-1.7, 18.5
		HU	HU		HU	HU
Absolute iodine concentration at 1.6 mg I/mL	6	1.9	1.7, 2.8	3	1.2	1.0, 1.5
		mg I/mL	mg I/mL		mg I/mL	mg I/mL

PC = Post contrast images

TUE = True unenhanced image

## Discussion

In our research, MDI and iodine quantification technique derived from DECT datasets demonstrated high accuracy in identifying internal enhancement in a renal lesion in which a mass can be distinguished from a high attenuation cyst. Moreover, the diagnosis performance of DECT parameters was similarly high as the attenuation measurement. Therefore, interpretation of a hyperattenuating renal lesion can be accurate without an aid of conventional attenuation difference if DECT datasets are available. As a result, TUE scanning can be omitted, leading to a reduction in the patient’s radiation exposure [4]. In this study, we only included a renal lesion with an attenuation between 10 and 70 HU at precontrast CT as definite simple cysts (attenuation less than 10HU) and definite hemorrhagic cysts (attenuation of 70 HU or higher at precontrast CT; defined as Bosniak Classification 2019 category II) rarely require enhancement assessment.

**Fig. 6:**
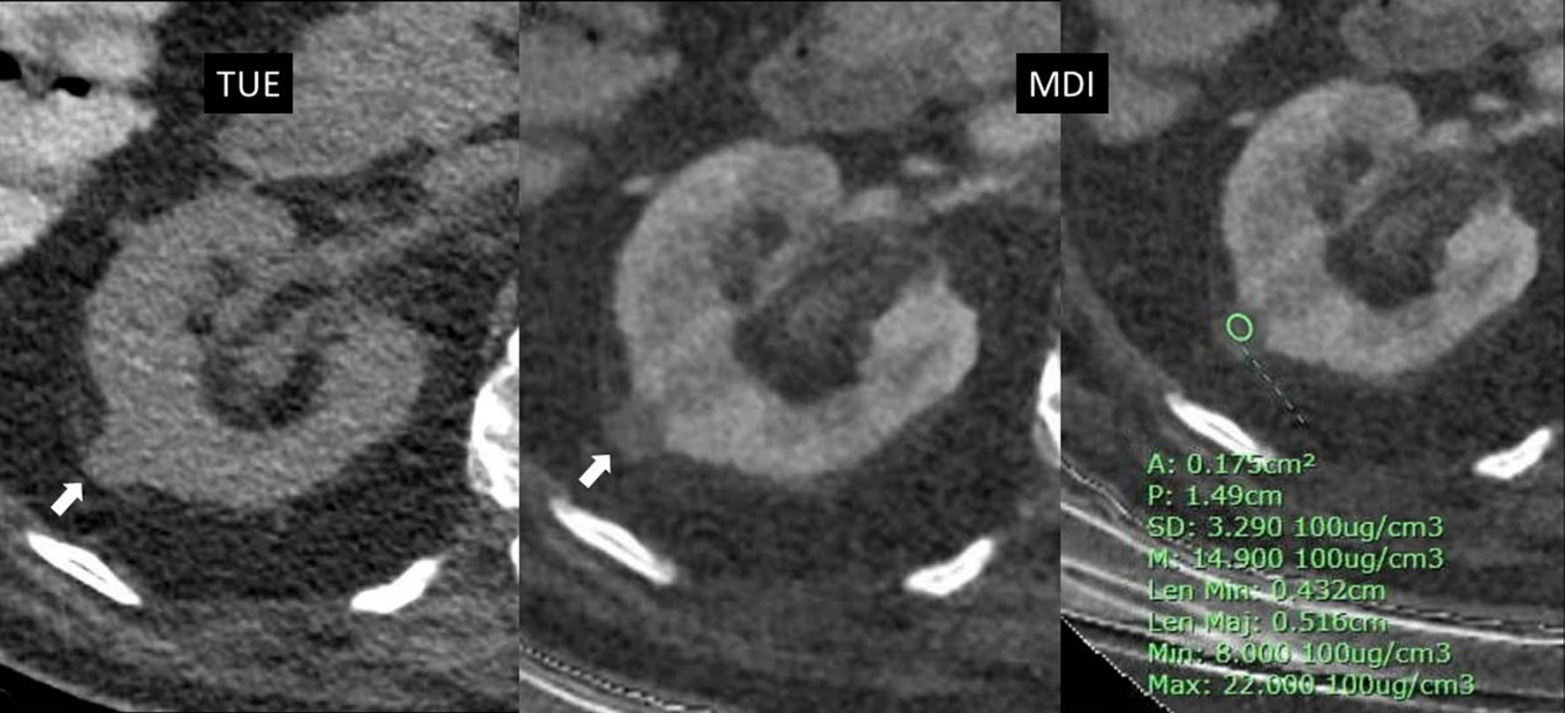
A 1.1 cm hyperattenuating cyst, measuring 20 HU on unenhanced imaging, with a slightly elevated absolute iodine concentration of approximately 1.49 mg I/mL. The mildly elevated iodine level still remained below the 1.6 mg I/mL threshold and no definite iodine density was observed on visual assessment. The is likely artifactual, possibly related to the small size of the lesion. TUE = True unenhanced image, MDI = Material density iodine images

In this study, we have also shown high diagnostic performance on image interpretation using MDI images either with MDI images alone or additional to conventional pre- and post-contrast images as it yielded high sensitivity of 94–97% and specificity of 82–94% for a renal mass characterization. We also demonstrated a strong to near complete interreader agreement and high diagnostic confidence of radiologists on image interpretation using MDI images. The prior study by Pourvaziri et al. evaluated a visual assessment using MDI images for renal lesion characterization and they found that MDI images enabled fast image interpretation due to high image quality and increased diagnostic confidence of readers irrespective of radiologists’ experience [6]. In that study, they also reported higher diagnostic accuracy of MDI images (AUC = 0.88) compared to that for subtraction conventional CT images (AUC = 0.75). These can imply that radiologist can confidently use MDI images either as stand-alone tools or as supplementary images as a quick tool for an initial overview of enhancement in a renal lesion then simplifying diagnosis as a mass or a cyst accordingly. When there is clearly no iodine density within a lesion, further evaluation is not required (Fig. 6). However, it is still crucial that evaluation of indeterminate renal lesion should not be solely rely on enhancement but a careful evaluation of morphologic features or correlation with other imaging modality such as US or MRI.

Iodine concentration derived from DECT datasets can be another effective tool for determination of enhancement in a renal lesion quantitatively. However, there are inherent platform-related differences and variability in iodine content thresholds in DECT. The study by Lennartz et al. demonstrated that inter-scanner differences in iodine quantification can be partially mitigated through normalization techniques, with the kidneys showing the lowest variability in iodine concentration across systems [10]. In this study using rsDECT, the absolute iodine concentration threshold of 1.6 mg I/mL was found to be optimal for distinguishing a mass and a hyperattenuating cyst with the sensitivity and specificity of 91% and 92% comparable to conventional method of pre- and post-contrast attenuation difference values. We also introduced the normalized iodine concentration threshold of 0.3 mg I/mL which achieved the sensitivity and specificity of 91% and 92%. These results are aligned with the prior studies [8, 10, 13, 15, 16]. In 2021, Mastrodicasa et al. determined whether a single-phase DECT could differentiate vascular and nonvascular renal lesions in the portovenous phase and found a similar performance of iodine concentration and attenuation measurements and a slightly higher performance of iodine concentration for differentiation of vascular lesions and hemorrhagic/proteinaceous cysts (AUC 0.87 vs. AUC 0.85, *p* < 0.001) [15]. In the study by Meyer et al., using iodine concentration for differentiating enhancing from nonenhancing renal lesions, the authors reported the optimized iodine quantification threshold of 2.0 mg I/mL for rsDECT and 1.0 mg I/mL for dual-source DECT and a combined optimized iodine quantification threshold of 1.4 mg I/mL [8]. Moreover, the prior study by Sadoughi et al., the authors found that no cysts were mistaken as enhanced lesions when an iodine concentration threshold of 1.2 mg I/mL with the rsDECT was used [10]. To differentiate benign and malignant renal lesions, the recent systemic review and meta-analysis showed that the iodine concentration threshold of 0.5 mg I/mL has an accuracy similar to the attenuation different threshold of 15HU and 20HU with a false positive rate of 0.04 [14]. In our study, using an iodine concentration threshold of 0.5 mg I/mL resulted in a very low specificity of 29% while using a higher iodine concentration threshold of 2 mg I/mL improved specificity to 97% but decreased sensitivity (71%), compared to the 1.6 mg I/mL threshold. A use of iodine density thresholds should be specific to the DECT platform. The diagnostic setting of iodine quantification measurements had been reported to be significantly higher for rsDECT than for other DECT platforms including dual-source DECT [8, 10, 13]. While the commonly used iodine quantification threshold of 0.5 mg I/mL is clinically feasible, however, our results support the use of a higher threshold with the specific rsDECT platform to enhance diagnostic accuracy in characterizing challenging renal lesions.

In this study, we reported 6.6% of cysts showing pseudoenhancement at the 1.6 mg I/mL iodine cut-off, which were considered false positives. This was a slightly higher rate than the 5.5% observed using a 20 HU attenuation difference cut-off on PC-TUE. However, the number of false-negative cases was similar between these two methods. Based on our observations, those cysts with pseudoenhancement on iodine quantification had an average size of 1.1 cm (range, 1.0–1.2 cm) and were located intraparenchymally (Fig. 6). Iodine quantification pseudoenhancement has been previously reported to artificially elevate iodine levels, particularly in small lesions surrounded by high-attenuation parenchyma [12]. This is also supported by the study by Meyer et al., which identified higher unenhanced attenuation and intraparenchymal lesion location as factors associated with both false positives and false negatives, likely due to incomplete correction of beam-hardening and image noise [8]. These findings may have important clinical implications, especially when evaluating small (approximately 1 cm) renal lesions, as pseudoenhancement of cysts may lead to lesion misclassification and result in unnecessary repeat imaging or treatment.

According to ACR guidelines and the Bosniak 2019 recommendations, contrast-enhanced CT or MRI with a dedicated multiphase protocol remains the standard for renal lesion characterization [17, 18]. However, DECT which is now widely adopted in clinical practice, has been shown to provide accurate characterization of renal lesions when a dedicated multiphase protocol is not available [17, 18]. Based on the high diagnostic performance of DECT with iodine quantification demonstrated in our study, the absence of measurable iodine content within a hyperattenuating renal lesion has the potential to indicate a benign etiology. This may propose a more conservative management approach and a complete assessment may not be required for such lesions.

There were some limitations in our study. First, the study carried a relatively small population size and retrospective nature which might have a selection bias and affected the generalizability of our findings. We did not have histopathological confirmation for all the lesions evaluated and the final diagnosis for some lesions relied on a combination of follow-up imaging. Although this is an imperfect reference standard, this approach is considered acceptable given the practical difficulty of obtaining histopathological confirmation for all renal lesions, which occur frequently in clinical practice. For visual assessment of MDI images, it was undeniably operator-dependent, however, we reduced this potential bias by measuring agreements between readers. Our study did not aim to distinguish between benign or malignant renal lesions but focused on differentiating enhancing versus non-enhancing features of renal lesions. This method might assist radiologists to deal with an incidentally renal lesion with an incomplete renal protocol. However, lesions with suspicious or enhancing features may still require a CT or MRI renal protocol for further characterization. Lastly, our study did not propose a tiered interpretation algorithm based on iodine thresholds for lesion characterization (e.g., cyst, mass, intermediate). Instead, we introduced a single iodine concentration cut-off point to differentiate between enhancing and non-enhancing lesions, with the goal of simplifying interpretation. However, the proposed tiered thresholds represent a potential direction for future research.

In conclusion, our study affirms that contrast-enhanced DECT with post-processing iodine images offers diagnostic performance equal to or better than attenuation measurements in assessing small (< 4 cm) hyperattenuating renal lesions, even in the absence of a dedicated renal CT protocol or true unenhanced images. This could lead to improved diagnostic workflows for renal lesion assessment using MDI and allow for the omission of TUE scanning in routine clinical practice, thereby reducing patient radiation exposure.

## Data Availability

No datasets were generated or analysed during the current study.

## References

[1] Silverman SG, Mortele KJ, Tuncali K, Jinzaki M, Cibas ES. Hyperattenuating renal masses: etiologies, pathogenesis, and imaging evaluation. Radiographics. 2007;27(4):1131–43. 17620471 10.1148/rg.274065147

[2] Schieda N, Davenport MS, Krishna S, Edney EA, Pedrosa I, Hindman N, et al. Bosniak Classification of Cystic Renal Masses, Version 2019: A Pictorial Guide to Clinical Use. Radiographics. 2022;42(1):E33. 34990332 10.1148/rg.219016

[3] Butaney M, Wilder S, Patel AK, Qi J, Mirza M, Noyes SL, et al. Initial Management of Indeterminate Renal Lesions in a Statewide Collaborative: A MUSIC-KIDNEY Analysis. J Urol. 2023;210(1):79–87. 36947795 10.1097/JU.0000000000003433

[4] Herts BR, Silverman SG, Hindman NM, Uzzo RG, Hartman RP, Israel GM, et al. Management of the Incidental Renal Mass on CT: A White Paper of the ACR Incidental Findings Committee. J Am Coll Radiol. 2018;15(2):264–73. 28651987 10.1016/j.jacr.2017.04.028

[5] Thiravit S, Brunnquell C, Cai LM, Flemon M, Mileto A. Use of dual-energy CT for renal mass assessment. Eur Radiol. 2021;31(6):3721–33. 33210200 10.1007/s00330-020-07426-z

[6] Pourvaziri A, Parakh A, Mojtahed A, Kambadakone A, Sahani DV. Diagnostic performance of dual-energy CT and subtraction CT for renal lesion detection and characterization. Eur Radiol. 2019;29(12):6559–70. 31134365 10.1007/s00330-019-06224-6

[7] Patel BN, Vernuccio F, Meyer M, Godwin B, Rosenberg M, Rudnick N, et al. Dual-Energy CT Material Density Iodine Quantification for Distinguishing Vascular From Nonvascular Renal Lesions: Normalization Reduces Intermanufacturer Threshold Variability. AJR Am J Roentgenol. 2019;212(2):366–76. 30667306 10.2214/AJR.18.20115

[8] Meyer M, Nelson RC, Vernuccio F, Gonzalez F, Schabel C, Mileto A, et al. Comparison of Iodine Quantification and Conventional Attenuation Measurements for Differentiating Small, Truly Enhancing Renal Masses From High-Attenuation Nonenhancing Renal Lesions With Dual-Energy CT. AJR Am J Roentgenol. 2019;213(1):W26-W37. 30917024 10.2214/AJR.18.20547

[9] Salameh JP, McInnes MDF, McGrath TA, Salameh G, Schieda N. Diagnostic Accuracy of Dual-Energy CT for Evaluation of Renal Masses: Systematic Review and Meta-Analysis. AJR Am J Roentgenol. 2019;212(4):W100-W5. 30714831 10.2214/AJR.18.20527

[10] Sadoughi N, Krishna S, Macdonald DB, Chatelain R, Flood TA, McInnes MDF, et al. Diagnostic Accuracy of Attenuation Difference and Iodine Concentration Thresholds at Rapid-Kilovoltage-Switching Dual-Energy CT for Detection of Enhancement in Renal Masses. AJR Am J Roentgenol. 2019;213(3):619–25. 31120787 10.2214/AJR.18.20990

[11] Lennartz S, Cao J, Pisuchpen N, Srinivas-Rao S, Locascio JJ, Parakh A, et al. Intra-patient variability of iodine quantification across different dual-energy CT platforms: assessment of normalization techniques. Eur Radiol. 2024;34(8):5131–41. 38189979 10.1007/s00330-023-10560-z

[12] Soesbe TC, Ananthakrishnan L, Lewis MA, Duan X, Nasr K, Xi Y, et al. Pseudoenhancement effects on iodine quantification from dual-energy spectral CT systems: A multi-vendor phantom study regarding renal lesion characterization. Eur J Radiol. 2018;105:125–33. 30017268 10.1016/j.ejrad.2018.06.002

[13] Kaza RK, Caoili EM, Cohan RH, Platt JF. Distinguishing enhancing from nonenhancing renal lesions with fast kilovoltage-switching dual-energy CT. AJR Am J Roentgenol. 2011;197(6):1375–81. 22109292 10.2214/AJR.11.6812

[14] Miron Mombiela R, Balschmidt T, Birch C, Lyngby CG, Bretlau T. Diagnostic performance of contrast enhancement to differentiate benign and malignant renal lesions in CT and MRI: a systematic review and meta-analysis of diagnostic test accuracy (DTA) studies. Abdom Radiol (NY). 2025;50(1):360–78. 39136719 10.1007/s00261-024-04514-2

[15] Mastrodicasa D, Willemink MJ, Madhuripan N, Chima RS, Ho AA, Ding Y, et al. Diagnostic performance of single-phase dual-energy CT to differentiate vascular and nonvascular incidental renal lesions on portal venous phase: comparison with CT. Eur Radiol. 2021;31(12):9600–11. 34114058 10.1007/s00330-021-08097-0 PMC9282667

[16] Marin D, Davis D, Roy Choudhury K, Patel B, Gupta RT, Mileto A, et al. Characterization of Small Focal Renal Lesions: Diagnostic Accuracy with Single-Phase Contrast-enhanced Dual-Energy CT with Material Attenuation Analysis Compared with Conventional Attenuation Measurements. Radiology. 2017;284(3):737–47. 28353408 10.1148/radiol.2017161872

[17] Expert Panel on Urologic I, Wang ZJ, Nikolaidis P, Khatri G, Dogra VS, Ganeshan D, et al. ACR Appropriateness Criteria(R) Indeterminate Renal Mass. J Am Coll Radiol. 2020;17(11S):S415-S28. 33153554 10.1016/j.jacr.2020.09.010

[18] Silverman SG, Pedrosa I, Ellis JH, Hindman NM, Schieda N, Smith AD, et al. Bosniak Classification of Cystic Renal Masses, Version 2019: An Update Proposal and Needs Assessment. Radiology. 2019;292(2):475–88. 31210616 10.1148/radiol.2019182646 PMC6677285

